# Isolation and Molecular Identification of Auxotrophic Mutants to Develop a Genetic Manipulation System for the Haloarchaeon *Natrinema* sp. J7-2

**DOI:** 10.1155/2015/483194

**Published:** 2015-05-21

**Authors:** Jie Lv, Shuai Wang, Yuchen Wang, Yuping Huang, Xiangdong Chen

**Affiliations:** ^1^State Key Laboratory of Virology, College of Life Sciences, Wuhan University, Wuhan 430072, China; ^2^Henan Academy of Medical and Pharmaceutical Sciences, Zhengzhou University, Zhengzhou 450052, China; ^3^Hubei Provincial Cooperative Innovation Center of Industrial Fermentation, Wuhan 430072, China

## Abstract

Our understanding of the genus *Natrinema* is presently limited due to the lack of available genetic tools. Auxotrophic markers have been widely used to construct genetic systems in bacteria and eukaryotes and in some archaeal species. Here, we isolated four auxotrophic mutants of *Natrinema* sp. J7-2, via 1-methyl-3-nitro-1-nitroso-guanidin mutagenesis, and designated them as J7-2-1, J7-2-22, J7-2-26, and J7-2-52, respectively. The mutant phenotypes were determined to be auxotrophic for leucine (J7-2-1), arginine (J7-2-22 and J7-2-52), and lysine (J7-2-26). The complete genome and the biosynthetic pathways of amino acids in J7-2 identified that the auxotrophic phenotype of three mutants was due to gene mutations in *leuB* (J7-2-1), *dapD* (J7-2-26), and *argC* (J7-2-52). These auxotrophic phenotypes were employed as selectable makers to establish a transformation method. The transformation efficiencies were determined to be approximately 10^3^ transformants per *µ*g DNA. And strains J7-2-1 and J7-2-26 were transformed into prototrophic strains with the wild type genomic DNA, amplified fragments of the corresponding genes, or the integrative plasmids carrying the corresponding genes. Additionally, exogenous genes, *bgaH* or *amyH* gene, were expressed successfully in J7-2-1. Thus, we have developed a genetic manipulation system for the *Natrinema* genus based on the isolated auxotrophic mutants of *Natrinema* sp. J7-2.

## 1. Introduction

Archaea were recognized as an independent domain distinct from eukarya and bacteria by Woese in the late 1970s [1, 2]. Unique biological features of archaea that help to adapt to harsh environment could provide opportunities to gain insight into a number of fundamental problems in biology. While so far, literatures about construction of genetic system in archaea are still relatively limited, in 1987, Cline and Ford Doolittle first demonstrated that* Halobacterium halobium* could uptake the naked genomic DNA of temperate halovirus *φ*H by PEG- (polyethylene glycol-) mediated spheroplast transfection method and produce plaques successfully [3], which shows the possibility to setup a genetic operation system for these organisms. To establish genetic manipulation system in archaea, selectable marker and shuttle vector based on the marker are critical. In terms of systems based on antibiotic resistance, mevinolin which inhibits 3-hydroxy-3-methylglutaryl coenzyme A (HMG-CoA) [4, 5] and novobiocin which inhibits DNA gyrase [6–8] are two main antibiotics that have successfully been applied in halophiles. In 1989, a resistance gene for mevinolin was isolated to construct the shuttle vector pWL102 for* E. coli *and* Haloferax volcanii*.* Hfx. volcanii *could be transformed with a shuttle vector by the PEG-mediated method with selection of mevinolin [9]. Later in 1990, shuttle vectors pUBP1 and pUBP2 were developed for the transformation of* Halobacterium halobium*, using the replicon region from halobacterial phage *φ*H and the mevinolin resistance as the selectable marker. Resistance gene for the gyrase inhibitor novobiocin was selected from the genus* Haloferax *and cloned into the endogenous plasmid pHK2 of* Haloferax *strain Aa2.2 to form pMDS2. And a shuttle vector pMDS1 was developed and efficiently transformed into* Hfx. volcanii *by using novobiocin resistance as a selectable marker [6, 7]. Some other vectors were also developed for these two halophiles; for example, pMPK35, pMPK52 based on pGRB1, pUBP4, and pUBP8 based on pHH1 [5] were developed for* Hbt. Halobium*; pWL101 based on pHV2 [4] and pMDS1 based on pHK2 [7] were developed for* Hfx. volcanii*. And, in 2010, a transformation system for* Pyrococcus furiosus *was described based on the shuttle vector system pYS2 from* Pyrococcus abyssi*, in which the* pyrE* gene was replaced by the HMG-CoA as a selectable marker conferring resistance to the antibiotic simvastatin [10]. Mayrhofer-Iro et al. developed a transformation/vector system for halo(alkali)philic members by using a genomic elements of* Natrialba magadii* virus *φ*Ch1 and a novobiocin resistance cassette as a selectable marker [11].

Besides the antibiotic resistance and shuttle vectors described above, auxotrophic markers are also useful selectable markers for construction of genetic system. Auxotrophs are a group of organisms that lost the ability to synthesize certain substances required for their growth owing to the presence of mutations. Compared to the wild type strain, the auxotrophic mutants cannot grow in minimal medium if the corresponding nutrients are not supplied. Auxotrophic strains have been widely employed for genetic investigation in bacteria and eukaryotes, but only a few cases have been documented in archaea. In 1987, several auxotrophic strains of* Methanococcus voltae* PS were obtained by irradiation with UV and gamma rays. These mutants were determined to be auxotrophic for histidine or purine and were used to demonstrate the occurrence of transformation with homologous wild type DNA by using the auxotrophic markers [12]. In 1990, auxotrophs of* Methanococcus maripaludis* created by ethyl methanesulfonate treatment were used to investigate the role of the acetyl-CoA pathway of autotrophic CO_2_ fixation in* Methanococcus maripaludis *[13]. Recently, a* Sulfolobus solfataricus pyrEF/lacS* mutant was used to construct a reporter gene system with integrative vectors containing the beta-galactosidase gene [14]. In the case of halophilic archaea, auxotrophy is an important tool since only limited selectable markers can be used in them. Several haloarchaeal auxotrophs obtained from mutagenesis or gene knockout have been employed in genetic studies. In 1985, for instance, auxotrophic mutants of* Hfx. volcanii* generated by chemical mutagenesis were used to demonstrate a native genetic transfer system in this extremely halophilic autotroph [15]. In 1990, Conover and Doolittle demonstrated that cosmid DNA prepared from* Escherichia coli* can be used to transform the histidine auxotroph* Hfx. volcanii* to a prototrophy [16]. Uracil auxotrophs based on the* pyrE*,* pyrF*, or* ura3* gene were later used to develop a gene knockout system for* Hfx. volcanii *[17, 18],* Halobacterium salinarum* [19],* Haloferax mediterranei*, and* Haloarcula hispanica *[20], respectively. Aromatic amino acid auxotrophy of* Hbt. salinarum* was obtained by in-frame deletions of OE1477R [21]. And, in 2014, Choi et al. reported a novel integrative expression vector which contained a* pyrE *gene as an auxotrophic selection marker for* Sulfolobus* species [22]. However, it should be noted that the employment of auxotrophic mutants is presently mainly confined to genetically tractable model organisms; thus similar research in halophiles is important for establishing their respective genetic manipulation system.

The halobacterial genus* Natrinema *gen. nov., which requires a medium with at least 10% salt concentration for cell growth (optimum 19.8%–25.1%), is a genus of halophilic archaea first proposed by McGenity et al. according to the phylogenetic analysis of 16S rRNA gene sequences and taxonomic properties [23]. This genus now contains seven species,* Natrinema altunense*,* Natrinema ejinense*,* Natrinema gari*,* Natrinema pallidum*,* Natrinema pellirubrum*,* Natrinema versiforme*, and* Natrinema salaciae*. Studies of the genus* Natrinema* have mainly focused on their phenotypic, physiological, biochemical, and taxonomic properties, and no studies analyzing their genetics or genetic engineering potential have yet been reported.

*Natrinema *sp. strains J7-1 and J7-2 were isolated from a salt mine in Yingcheng, Hubei Province, China [24]. The genomes of the two strains were sequenced (GenBank ID: PRJNA157873 and PRJNA89473), revealing that they were genetically identical excluding the fact that J7-1 contains a plasmid named pHH205 and J7-2 contains a plasmid named pJ7-I [25]. Interestingly, the 16,341 bp endogenous plasmid pHH205 included in J7-1 [24, 26] was proven to be the proviral genome of a temperate membrane-containing halophilic virus called SNJ1, which could be induced from J7-1 with mitomycin C and produces plaques on lawns of J7-2 [25, 27]. In addition, another provirus named SNJ2 has recently been found on the chromosomes of these two strains, but the susceptible host of this virus has not yet been identified (unpublished data). Thus the two strains could be valuable models to study the interaction of the proviruses and their host strains. These two strains have also been employed to conduct studies of the halophilic protease SptA [28, 29], heat shock protein 70 [30], and DNA fragments conferring promoter activity in three domains [31, 32]. However, the lack of genetic tools is delaying further studies of these strains. Fortunately, the availability of the genome sequence and the amino acid biosynthetic pathways of strain J7-2 [33] allow us to isolate and characterize auxotrophic mutants of these strains, which may be used to establish a genetic manipulation system for the genus* Natrinema*.

In the present study, we report the isolation of four auxotrophs of* Natrinema *sp. J7-2 via 1-methyl-3-nitro-1-nitroso-guanidin (NTG) mutagenesis. Their auxotrophic phenotypes and genes responsible for the phenotypes were determined. The auxotrophic phenotypes were further used to establish a DNA transformation protocol suitable for the strains. In addition, we showed that both the* bgaH* gene from* Haloferax alicantei* [34, 35] and* amyH *gene from* Har. hispanica* DSM 4426 [36, 37] could be integrated and expressed in strain J7-2-1 by using auxotrophy as a selectable marker.

## 2. Materials and Methods

### 2.1. Strains and Culture Conditions

Strains and plasmids used in this study are listed in Table 1.* Natrinema *sp. strains were grown in Halo-2 (H-2) medium prepared as previously described [27]. Modified growth media (MGM) contained 18% (w/v) salt water (SW) [38]; minimal medium (MM) used to isolate and identify* Natrinema *sp. J7-2 auxotrophs contained, per liter, 188 g NaCl, 43 g MgSO_4_·7H_2_O, 2.5 g KCl, 5 mM Tris HCl (pH 7.5), 5 mL 1 M NH_4_Cl solution, 5 mL 10% disodium succinic acid, 2 mL 0.05 M K_2_HPO_4_, 2 g glucose, 4.865 mL 1 M CaCl_2_, and 1 mL of trace element solution. The trace element solution contained, per 100 mL, 47 mg MnCl_2_·4H_2_O, 44 mg ZnSO_4_·7H_2_O, 5 mg CuSO_4_·5H_2_O, and 42 mg FeSO_4_·7H_2_O. If required, the corresponding amino acid was added to the MM medium to make a final concentration of 50 *μ*g mL^−1^. Solid agar was made with the addition of Bacto Agar (BD, USA) to the final concentration of 1.5%. Soluble starch was applied to the MGM and MM media at a final concentration of 1%-2%.

*E. coli* strains were grown in Luria-Broth (LB) medium supplemented with antibiotics when necessary [39]. Among them, DH5*α* was used for plasmid construction and propagation, and JM110 was employed to prepare unmethylated plasmid DNA for efficient transformation into* Natrinema *sp. J7-2.

### 2.2. Molecular Manipulation

Restriction endonuclease digestion, agarose gel electrophoresis, and additional molecular cloning techniques were carried out according to standard protocols [39, 40] or to the manufacturer's instructions. Small amounts of plasmid DNA were prepared using TIANprep Mini Plasmid Kit from TIANGEN (China). The primers used in this study are listed in Table 2. Restriction enzymes and other DNA modifying enzymes were purchased from TaKaRa or Fermentas and used according to the manufacturer's directions. Oligonucleotides were purchased from Sangon (Shanghai, China). DNA sequencing was performed by BGI (China).

### 2.3. Mutagenesis and Isolation of Auxotrophs from Strain J7-2

Strain J7-2 was grown in H-2 medium at 42°C for approximately 36 h. The cells were collected after centrifugation at 12,857 ×g for 2 min and resuspended in an equal volume of fresh medium. Mutagenesis was carried out using the following three methods: (1) NTG was added to the cell culture with final concentrations of 0.5, 0.75, 1.0, and 1.5 mg mL^−1^. The culture was kept at 37°C with shaking (180 rpm) for 1.5 h. (2) The concentrated cell cultures were directly spread on the solid H-2 plate. An NTG particle was then placed on the edge region of the plate. The plate was kept at 42°C until the appearance of the lawn. There was a large inhibition zone around the NTG particle. Cells collected from the edge of this zone were transferred to liquid H-2 medium and cultured for approximately 36 h at 42°C. (3) Under sterile conditions, the cell cultures were stirred using a magnetic stirrer under ultraviolet radiation (UV) for around 1.5 h. After the mutagenic treatment described above, six successive 10-fold dilutions of the cell cultures were taken. The 10^−4^, 10^−5^, and 10^−6^ dilutions of the cultures were spread on H-2 plates and incubated at 42°C for 7–10 days until colonies appeared. Subsequently, the colonies were inoculated in parallel on the MGM and MM plates and incubated at 42°C for 7–10 days. Strains that showed growth on MGM but failed to grow on the MM plate were considered as candidate auxotrophic mutants. The candidates were subjected to single colony isolation and inoculation in parallel on the MGM and MM plate experiments again, until the auxotrophic phenotype was confirmed.

### 2.4. Phenotypic and Molecular Characterization of NTG-Induced Auxotrophs

**Figure 1:**
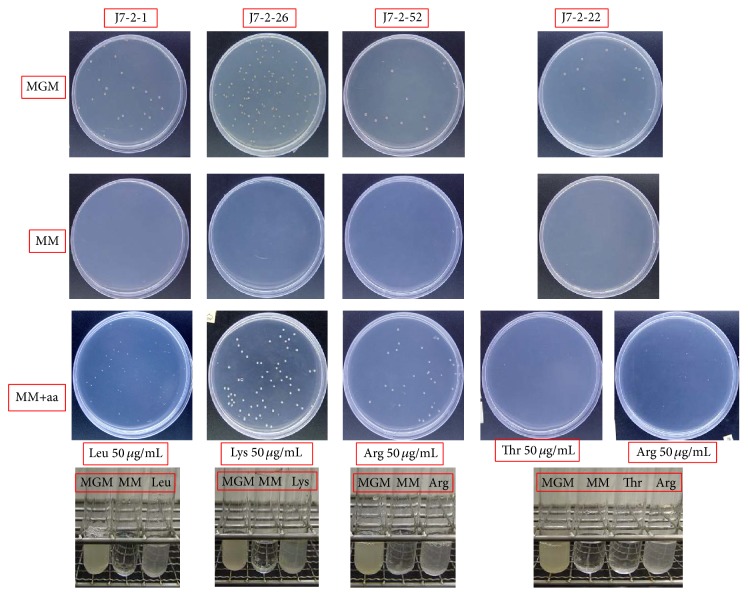
Growth behavior of the auxotrophic strains in different media. The method used to determine the nutrients required for the four auxotrophic mutants is described in Materials and Methods, after which the phenotypes of the mutants were confirmed. We picked out a single colony of each of the four mutants and cultivated it in a liquid MGM medium at 42°C. The cultures of each strain were obtained, centrifuged, and resuspended in MM medium. Then, the suspended cultures were inoculated in liquid media of MGM, MM, and MM with the corresponding nutrients added (as shown in the figure, leucine was added to the MM medium to reconfirm the phenotype of J7-2-1; lysine was added for reconfirming J7-2-26, arginine for J7-2-52, and threonine and arginine for J7-2-22). In corresponding solid media, the suspended cultures were diluted with MM media before spreading on the corresponding media plates. Their growth states were recorded and pictures were taken after storage at 42°C for 7–15 days. MGM: a complete medium that could support the growth of all J7-2 auxotrophic strains; MM: a minimal medium that could only support the growth of the J7-2 prototrophic strain; MM+aa: MM supplied with the relevant single amino acid to a final concentration of 50 *μ*g/mL.

To identify the phenotypes of the auxotrophic mutants, different nutrients (amino acids, vitamins, purine, and pyrimidine) were combined into different groups (Table 3). Auxotrophic mutants were spread on the MM plates. Small filter papers (approximately 5 mm in diameter) soaked in different types of liquid nutrition were placed on separate regions of the agar surface. Filter paper soaked with MGM medium was used as control. The plates were kept at 42°C for 7–15 days. According to their growth status, the nutrients required for the growth of the auxotrophic mutants were determined. Subsequently, the auxotrophic mutants were cultured again in both solid and liquid media of MGM, MM, and MM with added nutrients to reaffirm their phenotypes (Figure 1).

To find significant mutations in the auxotrophic mutants, genes important in the pathways of the corresponding amino acid synthesis were analyzed by PCR and DNA sequencing. The mutant genes were determined by comparison of the corresponding DNA sequences between the wild type strain and the auxotroph. Information about these genes and their primers is shown in Tables 2 and 4.

### 2.5. Isolation of* Natrinema *sp. J7-2 Total Genomic DNA

High molecular weight* Natrinema *sp. J7-2 genomic DNA was prepared according to the protocol of TIANamp Bacteria DNA Kit from TIANGEN (China). DNA samples prepared were examined by agarose gel electrophoresis and then used directly for transformation.

### 2.6. Transformation

All procedures were performed at room temperature unless indicated otherwise. The main transformation process of the auxotrophic mutants of* Natrinema *sp. J7-2 was performed based on the transformation protocol used for* Hfx. volcanii* [41] with several modifications: the standing time was modified from 10 min to 15 min to form spheroplasts; regeneration time was altered from 20–30 min at room temperature (RT) to 2–5 hours at 37°C and the transformation solutions were changed from 1 M NaCl to 2 M NaCl, a higher saline concentration suitable to* Hbt. salinarum *[41]. Finally, the resultant mixture was spread on the selected solid medium and stored at 45°C for 7–10 days to detect the transformants.

### 2.7. Molecular Identification of the Transformants

Target gene fragments were amplified from the genomic DNA of the wild type strain, auxotrophic strain, and the corresponding transformant. The DNA sequences were comparatively analyzed to determine whether the mutation sites had been complemented by the transformation.

### 2.8. *β*-Galactosidase Detection

The *β*-galactosidase activity of* Natrinema *sp. J7-2-1 transformants on plates was determined as described previously [44]. Briefly, agar plates were sprayed with X-gal (20 mg mL^−1^); blue colonies indicated the existence of *β*-galactosidase activity.

### 2.9. Amylase Activity Assay

The amylase activity of* Natrinema *sp. J7-2-1 transformants on plates was determined as described previously [37, 44] using 18% MGM supplemented with 2% (w/v) soluble starch. After incubation at 42°C for 5–7 days, plates were flooded with 0.3% I_2_/0.6% KI solution; a clear zone around the growth indicated starch hydrolysis.

## 3. Results and Discussion

### 3.1. Isolation of Auxotrophic Mutants of* Natrinema* sp. J7-2 by Mutagenesis

The halophilic archaea* Natrinema* sp. J7-2 could be a useful model to investigate the interactions between provirus and host, but such studies have so far been prevented by a lack of genetic tools. As genetic manipulation systems based on auxotrophic selectable markers have been developed in other halophilic archaea, such as* Hb. salinarum NRC-1*,* Haloferax mediterranei*, and* Haloarcula hispanica*, we decided to isolate auxotrophic mutants of* Natrinema* sp. J7-2 and develop a genetic system based on them.

Before mutagenesis,* Natrinema* sp. J7-2 was tested that it could grow on MM plate (Figure S1 in Supplementary Material available online at http://dx.doi.org/10.1155/2015/483194). The cultures of* Natrinema* sp. J7-2 having undergone NTG or UV mutagenesis were spread on the MGM plate to obtain individual colonies; randomly selected colonies were inoculated on the MGM and MM plates in parallel. Colonies which appeared on the MGM plates but not on the corresponding MM plates were considered as candidate auxotrophic mutants. Ten colonies from approximately 7000 randomly selected candidates were able to grow on the MGM plates, but not on the corresponding MM plates. These candidate auxotrophic mutants were purified using the spread plate method on MGM agar media, and the resultant individual colonies were again inoculated on the MGM and MM plates in parallel for further identification. Finally, four auxotrophic mutant strains were confirmed and were named as J7-2-1, J7-2-22, J7-2-26, and J7-2-52 (Figures S2 and S3). All were obtained from the NTG (0.75 mg mL^−1^) mutagenesis treatment, which has previously been used to isolate mutants in* Sulfolobus acidocaldarius* [45] and other species [46, 47].

### 3.2. Phenotypic and Molecular Characterization of Auxotrophic Mutants of* Natrinema* sp. J7-2

The phenotypes of the isolated auxotrophic strains were determined by restoration of their growth on the MM plate by supplying them with a range of nutrient combinations (as shown in Tables 3(a), 3(b), 3(c), and 3(d)). The results indicate that all were auxotrophic for specific amino acids. As shown in Table 3(e), strain J7-2-1 was a leucine auxotroph, since it could grow in MM medium supplied with groups ALL, 1, A, or H, all of which contain leucine. Strain J7-2-26 was determined to be a lysine auxotroph because it exhibited growth only on MM plates supplied with groups ALL, 1, B, or H, all of which contain lysine. Strain J7-2-52 was an arginine auxotroph as it could grow in MM medium supplied with groups ALL, 1, B, or F, all of which contain arginine. However, strain J7-2-22 could be auxotrophic for either arginine or threonine (Table 3(e)) because it could grow not only in MM medium supplied with groups ALL, 1, B, or F, but also on MM plates supplied with group I. The auxotrophic phenotypes of the four mutant strains were confirmed by assessment of their ability to grow in MM medium supplied with the corresponding single amino acid (Figure 1). The results reaffirmed that strain J7-2-1 is auxotrophic for leucine, strain J7-2-26 is auxotrophic for lysine, and both strains J7-2-22 and J7-2-52 are auxotrophic for arginine.

After the phenotypes of the auxotrophic strains were determined, we wanted to identify the mutations responsible for them. Each of the genes predicted to be involved in the corresponding amino acid biosynthesis was amplified from the genomes of the auxotrophs and the wild type strain (Figure S4). The mutation sites were determined by comparative analysis of the gene sequences (Table 4). The results showed that strain J7-2-1 (Leu^−^) has a 12 bp fragment deletion from 690 bp to 701 bp in* leuB*, whereas strain J7-2-26 (Lys^−^) has an 18 bp fragment deletion from 583 bp to 600 bp in* dapD *(Figure S5). We believe that these deletions might be the cause of their auxotrophic phenotypes. Consistent with its arginine auxotrophic phenotype, a single nucleotide substitution at position 1051 bp of the* argC* gene was identified in strain J7-2-52. This substitution resulted in a change of the codon from GGG to GAG. However, no mutations were identified in the coding regions of the genes involved in arginine biosynthesis in the other arginine auxotroph strain J7-2-22, indicating that its phenotype might be the result of mutations in other regulatory genes for arginine biosynthesis.

### 3.3. Establishment of a Genetic Transformation System for the* Natrinema* Genus

In 1987, Cline and Ford Doolittle developed a PEG-mediated transformation protocol for* Hfx. volcanii* [3]. In this protocol, cells are first treated with EDTA, which converts the cell to a spheroplast by removing its paracrystalline glycoprotein surface layer. DNA is then introduced into the spheroplasts with the help of PEG600, after which the cells recover in a regenerate solution or rich media before plating on a selective medium [48]. Although this transformation protocol has been used in a number of archaea, especially haloarchaea, it is only applicable for species in which a spheroplast can be generated readily [49].

**Figure 2:**
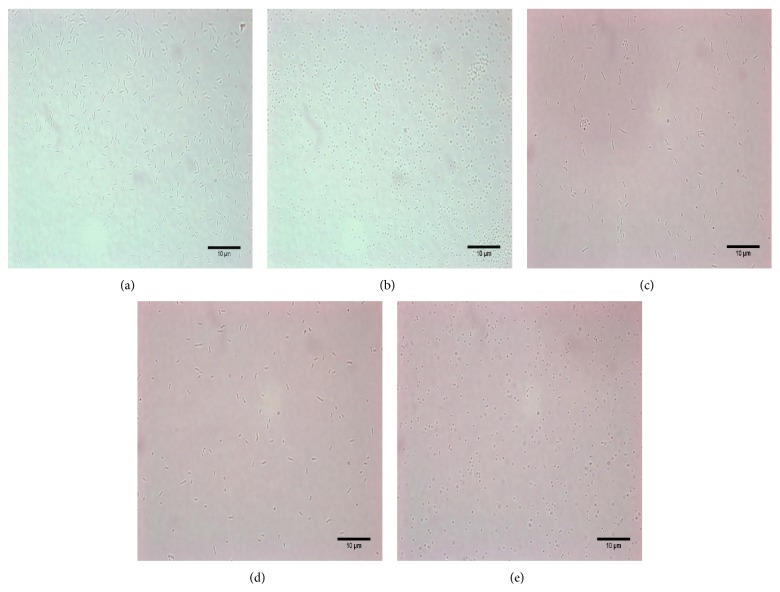
Morphological changes to the cells during the transformation process. As there was no transformation protocol available for the genus of* Natrinema*, we used the transformation process used for* Hfx. volcanii*. All procedures were performed at room temperature. We picked out a single colony of J7-2 and cultivated it in liquid MGM media at 42°C. Selected cultures of J7-2 were obtained and treated with spheroplasting solution for 15 min, while some were not treated to provide a control. Cell morphology was observed and recorded under the microscope. The results are shown in (a) and (b). During the recovery process, three different regeneration solutions were tested to determine whether the cell morphology could be recovered to short rods and which medium was more suitable for* Natrinema*: H-2 medium, 18% MGM and regeneration solution. We modified the protocol slightly. The regeneration time was changed from 20–30 min at RT to 2 to 5 hours at 37°C and the transformation solutions were also changed from 1 M NaCl to 2 M NaCl. The regeneration results were also observed and recorded under microscope. All recovered results are shown in (c), (d), and (e). (a) Cells of J7-2 grown in MGM medium before spheroplasting solution treatment. (b) Cells of J7-2 treated with spheroplasting solution. (c) Cells of J7-2 regenerated with H-2 medium treatment. (d) Cells of J7-2 regenerated with 18% MGM medium treatment. (e) Cells of J7-2 regenerated with regeneration solution treatment.

As there was no transformation protocol available for the* Natrinema *genus, we aimed to develop a transformation method for* Natrinema *based on the above transformation protocol for* Hfx. volcanii*. We determined whether* Natrinema* J7-2 cells could become spheroplasts after EDTA treatment and secondly if the spheroplast could recover in regenerate solution. The standing time in the process was modified to 15 min to form spheroplasts; regeneration time was changed as two to five hours at 37°C and the transformation solutions were also changed to 2 M NaCl, a higher saline concentration suitable for* Hbt. salinarum*. As shown in Figures 2(a) and 2(b) and Table 5, the morphology of the cells changed from rod shaped to spherical after EDTA treatment for 15 min, indicating the generation of the spheroplast. Three different regeneration solutions were tested to identify the most suitable for recovery of* Natrinema *spheroplast: H-2 medium; 18% MGM; and regeneration solution. The first two are rich media used to cultivate* Natrinema *sp. J7 serials strains and* Hfx. volcanii*, respectively. The regeneration solution, comprised of an 18% salt solution supplied with 15% sucrose, 0.05% yeast extract, 0.01% peptone, and 0.1% casamino acids, was used for transformation of* Hfx. volcanii*. Cell morphology could be restored to rods using all three mediums, but the cells recovered more effectively in the H-2 medium and 18% MGM than in the regeneration solution (Figures 2(c), 2(d), and 2(e) and Table 5). Thus, in the following experiments, we chose H-2 medium as the recovery solution.

**Figure 3:**
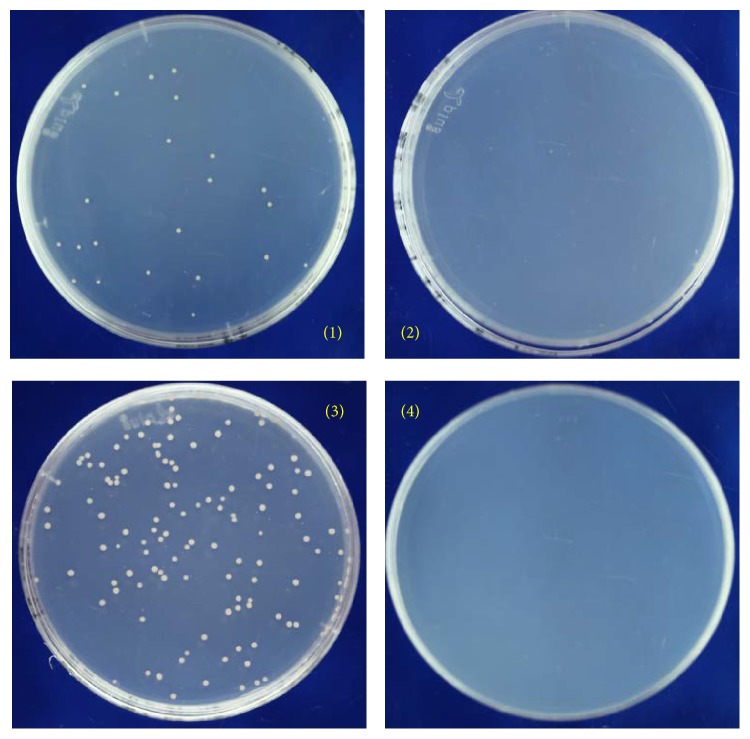
Results of the transformation of J7-2-1 and J7-2-26 with J7-2 genome. The wild type* Natrinema* sp. J7-2 total genomic DNA was isolated and then transformed into two auxotrophic mutants, J7-2-1 and J7-2-26. The transformation mixture was spread on the MM plates and cultivated at 42°C for 15 days. The detailed transformation process was described in Materials and Methods. (1) J7-2-1 transformed with J7-2 genome. (2) Negative control, J7-2-1 transformed with salt solution without DNA. (3) J7-2-26 transformed with J7-2 genome. (4) Negative control, J7-2-26 transformed with salt solution without DNA.

Since the spheroplast of* Natrinema* could be generated and recover successfully, we tested if spheroplasted J7-2 cells could be transformed by exogenous DNA using the isolated auxotrophic mutants J7-2-1 and J7-2-26 as recipients. The genomic DNA of* Natrinema *sp. J7-2 was prepared as the transforming DNA and applied to auxotrophic strains. As strains J7-2-1 and J7-2-26 require the presence of leucine or lysine for growth, only recombinants that restore the ability to synthesize leucine or lysine would grow on MM plates. As shown in Figure 3, strains of J7-2-1 and J7-2-26 transformed with the genomic DNA of J7-2 appeared in the MM plates (plates 1 and 3), but colonies did not appear on plates when DNA was not added to the spheroplasts of J7-2-1 and J7-2-26. The transformation efficiency is estimated to be 3.62 × 10^3^ transformants per *μ*g DNA. To confirm the results, the* leuB* gene of the transformants of J7-2-1 and the* dapD* gene of the transformants of J7-2-26 were amplified and subjected to sequencing. Compared to the parent strains (J7-2-1 has a 12 bp fragment deletion from 690 to 701 bp in* leuB *gene and J7-2-26 has an 18 bp fragment deletion from 583 to 600 bp in* dapD *gene), the results showed that the mutations had been corrected in all the transformants, presumably by homologous recombination (Figures S5 and S6). These results indicate that, using the protocol we developed, exogenous DNA can be transformed into* Natrinema* and the resultant auxotrophic strains represent useful genetic tools.

To confirm that the auxotrophs were transformed to prototrophs by homologous recombination at the corresponding mutated genes, the* leuB* gene fragment and the* dapD* gene fragment from the genome of the wild type strain were amplified and transformed into J7-2-1 and J7-2-26 spheroplasts, respectively. As expected, transformants of each auxotroph transformed with the matching gene appeared on the MM plate and sequencing of* leuB* or* dapD* from the corresponding transformants confirmed that the mutation had been corrected (Figure S6). Furthermore, in the J7-2-1 transformation process, we amplified two fragments, KM1752, a* leuB* cloning fragment containing the promoter from J7-2, and HM1752, a* leuB* open reading frame (ORF) cloning fragment from J7-2 (Table 1), to identify any difference in transformation result. No obvious difference was observed (Figure S6). It is noteworthy that although the amount of* leuB* or* dapD* coding fragment should be much higher when transformed with the amplified gene fragment than that of transformation with genomic DNA, transformation efficiency of the former was slightly lower (1.02 × 10^3^ transformants per *μ*g DNA versus 3.62 × 10^3^ transformants per *μ*g DNA). We speculate that this is because the amplified fragments were short in length, resulting in a reduced efficiency of homologous recombination. However additional evidence is needed to prove this hypothesis.

### 3.4. Possibility of Transformation with Circular Plasmids and the Ability to Express Exogenous Genes in Auxotrophic Strains through Homologous Recombination

The above results showed that auxotrophs of* Natrinema *sp. J7-2 can be transformed to prototrophs by transformation with genomic DNA or amplified gene fragments of the wild type strain. However, both of them were linear DNA fragments. We wanted to know whether circular plasmids could also be transformed. Three plasmids containing the corresponding genes were constructed and named pUC19-KM1752, pUC19-HM1752, and pUC19-HM4049 (Table 1). The former two plasmids carrying the* leuB* gene were transformed into auxotrophic strain J7-2-1, while the latter, carrying the* dapD* gene, was transformed into auxotrophic strain J7-2-26. They were subsequently demethylated in* E. coli *strain JM110. As expected, all three circular plasmids could be transformed efficiently into auxotrophs and the prototrophic phenotype was restored on MM plates. We further quantified transformation efficiency, which was equal to approximately 2.96 × 10^3^ transformants per *μ*g DNA. Although the transformation efficiency is slightly increased, compared with the amplified gene fragment, it is still relatively low compared to that of* Hfx. volcanii *(10^3^ transformants per *μ*g DNA versus 10^6^ transformants per *μ*g DNA [7]). One possibility is that the transformation method we developed still requires improvement, though it is also possible that* Natrinema* sp. J7-2 is less effective than* Hfx. volcanii* at taking up extracellular DNA and performing homologous recombination. Nevertheless, the successful transformation of DNA into the auxotrophic strains of J7-2 shows that transformation in* Natrinema* is possible and the* leuB* and* dapD* genes could be used as selectable markers for the development of genetic manipulation systems.

**Figure 4:**
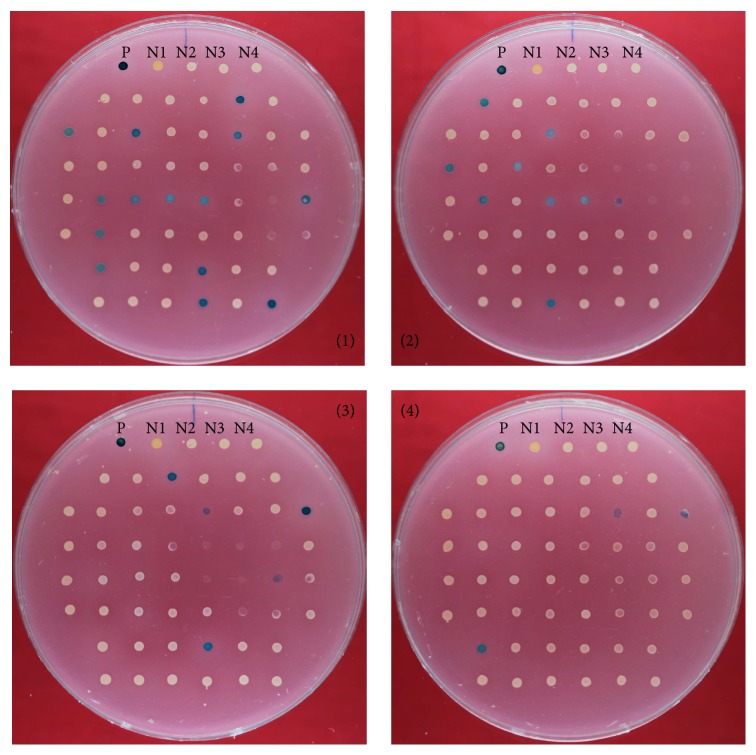
Expression of* bgaH* gene in J7-2-1 via the integrative plasmid pUC19-HM1752-*bgaH*. The plasmid pUC19-HM1752-*bgaH* containing* bgaH* was transformed into strain J7-2-1. In total, 200 transformants were picked out randomly and cultured. The activity of *β*-galactosidase in these 200 transformants was detected on four MGM plates (50 transformants on each plate) supplied with X-gal (detailed process is discussed in Materials and Methods). If the transformants had *β*-galactosidase activity, the color of the corresponding colony would turn blue. The results were recorded and pictures taken after the four plates were stored at 42°C for 7–15 days. P: positive control. N1: negative control* Hfx. volcanii *DS52. N2: negative control J7-2-1. N3: negative control J7-2-1 transformed with J7-2 genome. N4: negative control J7-2-1 transformed with KM1752 fragment.

Sequencing of the genomic regions of the transformants of J7-2-1 or J7-2-26 revealed that the mutated* leuB* or* dapD* genes had been replaced with wild type* leuB* and* dapD *(Figure S6), suggesting that the restoration of growth on the MM plate was due to homologous recombination between the chromosome and the incoming plasmids. Since the circular plasmids could be transformed into auxotrophs, we attempted to use these plasmids to integrate and express exogenous genes in the auxotroph strain J7-2-1. The* bgaH* gene from* Hfx. alicantei* [34, 35] was cloned into pUC19-HM1752 to produce the plasmid pUC19-HM1752-*BgaH*, which was then transformed into J7-2-1 and selected on MM plates. The resultant transformants were analyzed for the expression of the *β*-galactosidase using MGM plates supplied with X-gal. As shown in Figure 4, 31 of 200 tested transformants (ca. 15.5% of the transformants) displayed *β*-galactosidase activity, detected by a color change (from pink to blue), on the MGM plates supplied with X-gal.

**Figure 5:**
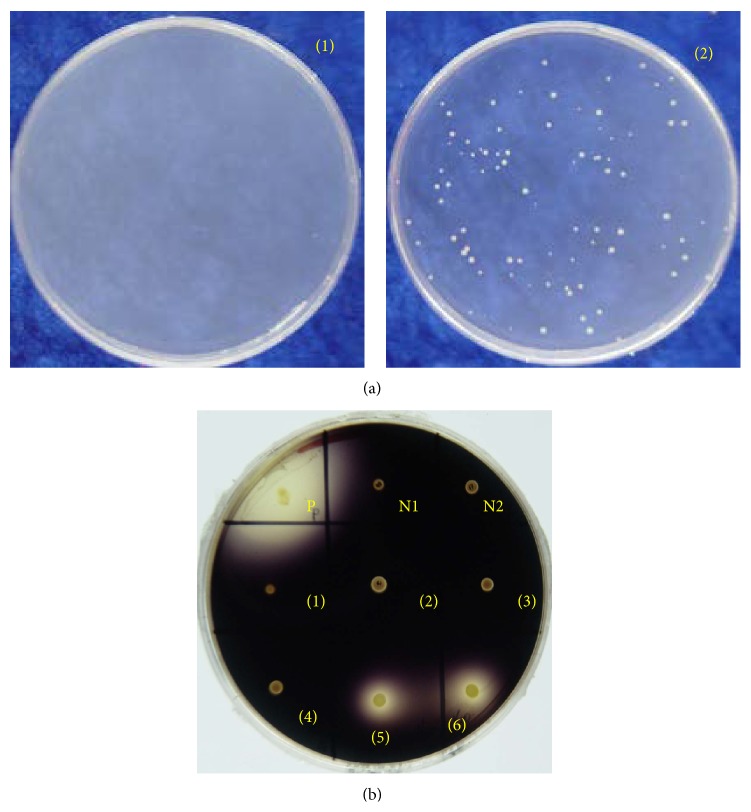
Expression of* amyH* gene in J7-2-1 via the integrative plasmid pUC19-HM1752-*amyH*. The plasmid pUC19-HM1752-*amyH* containing* amyH* was transformed into strain J7-2-1. Twenty-nine transformants were picked out randomly and cultured. Amylase activity in these transformants was detected on an MGM plate supplied with 2% (w/v) soluble starch. (detailed process is discussed in Materials and Methods). The plate was flooded with 0.3% I_2_/0.6% KI solution after incubation at 42°C for 7–15 days. If the transformants had amylase activity, a clear zone indicating starch hydrolysis would be observed. The results were recorded and pictures were taken. (a) (1) Negative control, J7-2-1 transformed with salt solution without DNA. (2) J7-2-1 transformed with integrative plasmid pUC19-HM1752-*amyH*. (b) MGM plates containing soluble starch are used to detect the expression of* amyH *gene in J7-2-1 by integrative plasmid pUC19-HM1752-*amyH*. P. Positive control. N1: negative control* Haloferax volcanii *DS52. N2: negative control J7-2-1. N3: negative control J7-2-1 transformed with J7-2 Genome. N4: negative control J7-2-1 transformed with KM1752 fragment. 1–29: samples from transformants of J7-2-1 transformed with integrative plasmid pUC19-HM1752-*amyH*.

Besides the* bgaH* gene from* Hfx. alicantei*, we also tested the expression of the* amyH *gene from* Har. hispanica *DSM 4426 [36] in strain J7-2-1. Similar to* bgaH*,* amyH* was cloned into plasmid pUC19-HM1752 and the resulting plasmid pUC19-HM1752-*amyH* was transformed into J7-2-1. Transformants were screened on MM plates (Figure 5(a)). Twenty-nine transformants were selected and inoculated on the MGM plate containing soluble starch in order to detect the expression and activity of the* amyH* gene. As shown in Figure 5(b), four out of the 29 transformants exhibited amylase activity and produced a hydrolysis ring on the MGM plate. Consistent with this, the* amyH *gene could be amplified from two randomly selected transformants that produced the hydrolysis ring, and sequence analysis confirmed that the* amyH *gene was integrated into the genome by a recombination event at the* leuB *gene. Other elements on the integrative plasmid, such as the replication region, the antibiotic gene of ampicillin, and the* lacZ *gene in pUC19, were also detected by PCR amplification from the same two randomly selected transformants conferring amylase activity (Figure S7). Interestingly, we found that specific fragments within the ampicillin resistance gene and* ColE1 *replication region could be detected in a randomly selected transformant without amylase activity. Currently, it is unclear why the* amyH* gene was dropped off from the chromosome; one possibility is that some sections of pUC19-HM1752-*amyH* were integrated into this transformant, but not including the* amyH *gene or the* lacZ *gene. Another possibility is that a secondary recombination event occurred between the J7-2-1 genome and the integrative plasmid, resulting in regions including* amyH *gene or* lacZ *gene dropping off from genome. Additional studies are required to solve this problem and therefore increase the percentage of positive transformants.

**Figure 6:**
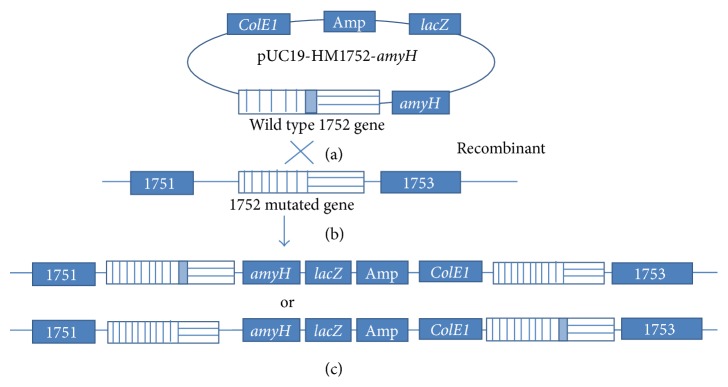
The possible integration position of exogenous DNA in the genome of transformants conferring amylase activity. Strain J7-2-1 was transformed with pUC19-HM1752-*amyH* and the schematic diagram shows the possible crossover event between the plasmid and J7-2-1 genome. (a) Plasmid pUC19-HM1752-*amyH*. (b) J7-2-1 genome with the mutated 1752 gene. (c) The possible integration position of exogenous DNA on the genome of the transformants conferring amylase activity.

The integration relative positions of the exogenous* amyH *gene on the genomes of transformants conferring amylase activity were identified through amplification experiments and sequencing with different primers and different primer combinations in the plasmid and are shown in Figure 6, confirming that the plasmid was integrated into the chromosome at the* leuB* locus by homologous recombination.

Nonetheless, the auxotrophs isolated here have been proven to be useful as selectable markers for the expression of exogenous genes in* Natrinema*. These auxotrophs could also facilitate the development of more sophisticated genetic tools to study other proteins and their functions in the genus of* Natrinema* and to investigate horizontal gene transfer between strains from this genus and others. In ongoing research, we are attempting to use the elements of the halophilic virus SNJ1 to construct a shuttle vector system for* Natrinema *sp. J7-2 and CJ7 (another* Natrinema *strain without any plasmid). We believe that the above provide useful information for further study and will contribute to understanding the genetics of* Natrinema *genus and halophilic archaea.

## Supplementary Material

Supplementary Figure S1: showed the growth of Natrinema sp. J7-2 on MM plate.
Supplementary Figure S2: showed that in the isolation process of mutants, four strains J7-2-1, J7-2-22, J7-2-26 and J7-2-52 which grow on MGM but failed on MM plate were obtained.
Supplementary Figure S3: the auxotroph of the four auxotrophic strains were reconfirmed with the same experimental procedure that they grow on MGM but failed on MM plate.
Supplementary Figure S4: amplification of important genes in the pathways of the amino acid synthesis from the four auxotrophic strains were shown.
Supplementary Figure S5: showed that the molecular characterization of the gene mutation status of the auxotrophic strains.
Supplementary Figure S6: showed the complementation of the mutation genes by auxotrophic strains' transformation and comparison the genes of these different transformants.
Supplementary Figure S7: amplification of specific fragments in J7-2-1/ pUC19-HM1752-amyH transformants conferring amylase activity were shown.

## Figures and Tables

**Table 1:** Strains, plasmids, and amplification fragments used in this work.

Strains, plasmids, and amplification fragments	Description	Source or reference
Strain		
*Natrinema *sp. J7-2	Haloarchaeal strain isolated from a salt mine	CCTCC AB91141, China [24, 25, 27]
*Natrinema *sp. J7-2-1	Auxotrophic strain from J7-2, Leu^−^	This work
*Natrinema *sp. J7-2-22	Auxotrophic strain from J7-2, Arg^−^	This work
*Natrinema *sp. J7-2-26	Auxotrophic strain from J7-2, Lys^−^	This work
*Natrinema *sp. J7-2-52	Auxotrophic strain from J7-2, Arg^−^	This work
*Escherichia coli* DH5*α*	*F* ^−^ *endA1 glnV44 thi-1 recA1 relA1 gyrA96 deoR nupG *Φ*80dlacZ*Δ*M15 *Δ*(lacZYA-argF)U169, hsdR17(r*_*K*_^−^ *m*_*K*_^+^ *), λ*−	[42]
*Escherichia coli* JM110	*rpsL thr leu thi lacY galK galT ara tonA tsx dam dcm glnV44 *Δ*(lac-proAB) e14-[F*′* traD36 proAB* ^+^ * lacI * ^*q*^ *lacZ*Δ*M15] hsdR17(r*_*K*_^−^ *m*_*K*_^+^)	[43]
Plasmid		
pUC19	Cloning vector	Lab stock
pMD19T-simple	Sequence vector	Purchased from TaKaRa
pUC19-KM1752	pUC19 containing* leuB *gene with* Kpn*I and* Mfe*I from J7-2	This work
pUC19-HM1752	pUC19 containing the ORF of *leuB *gene with *Hind*III and *Mfe*I from J7-2	This work
pUC19-HM4049	pUC19 containing *dapD* gene with *Hind*III and *Mfe*I from J7-2	This work
pUC19-HM1752-*amyH *	pUC19-HM1752 containing *amyH* gene from *Haloarcula hispanica* DSM 4426	This work
pUC19-HM1752-*bgaH *	pUC19-HM1752 containing *bgaH* gene from *Haloferax alicantei *	This work
Amplification fragments		
KM1752	*leuB* gene containing promoter from J7-2 with *Kpn*I and* Mfe*I	This work
HM1752	Amplification fragments of *leuB* gene from J7-2 with *Hind*III and* Mfe*I	This work
HM4049	Amplification fragments of *dapD* gene from J7-2 with *Hind*III and* Mfe*I	This work

**Table 2:** Primers used in this work.

Primer name	Sequence (5′-3′)	Restriction site
*Kpn*I-1752	TAAGGTACCTCGCTGCGATCTTGCAGGGACT	*Kpn*I
1752-*Mfe*I	TAACAATTGCGGCGGTCGCGGGCCACTAAA	*Mfe*I
*Hind*III-1752	TTAAAGCTTATGACTCACGAAATCGCCGTCATT	*Hind*III
*Hind*III-4049	TTAAAGCTTATGAGCGCACTCGAAA	*Hind*III
4049-*Mfe*I	TATCAATTGCCGGTTACGCCACCCCGCG	*Mfe*I
*MfeI*-*amyH *	AATCAATTGATCATATATGCCAACTATTTA	*Mfe*I
*amyH*-*Pst*I	AAACTGCAGTAGTGGAAAGCGAGCCAGC	*Pst*I
*leuA*-F	ATGGCACGTTATCCGCCATCT	*/ *
*leuA*-R	TCAGTCGTCGGCCGGCGT	*/ *
*leuC*-F	ATGAGCGCGGAAACGCTG	*/ *
*leuC*-R	TCATACCGAGGTCACCTCCT	*/ *
*leuD*-F	ATGACGGACGAGGTCGAGATT	*/ *
*leuD*-R	TACTCCGCTTCGGGAATCGC	*/ *
*leuB*-F	GTGAGTATGACTCACGAAAT	*/ *
*leuB*-R	TTACAGTCGGTCGATGAT	*/ *
*dapA*-F	GCGACCCAAGAGACCACA	*/ *
*dapA*-R	TCATCGCTCATCCTCCG	*/ *
*dapF*-F	ATGAATATCCCATTTCAGAAGT	*/ *
*dapF*-R	TCACGGCTCGGCCGGGGATT	*/ *
*lysA*-F	ATGAACGAAACCCAAACGTT	*/ *
*lysA*-R	TATCGGTCGTATTCGTTGTC	*/ *
*dapD*-F	ATGAGCGCACTCGAAACCGA	*/ *
*dapD*-R	TTACTCCCGCAGCGCGTCCT	*/ *
*dapB*-F	ATGACGACGCGAATCGGCGTCAC	*/ *
*dapB*-R	TCATTCGCTGATCACGTCTGCGAA	*/ *
*argF*-F	ATGACGACCGATTCCGATCC	*/ *
*argF*-R	TCACTCGAGCACCCAACTCA	*/ *
*argE*-F	ATGAGTGCAACCATGGACGACA	*/ *
*argE*-R	TCATTCGTCGTCCTCGCGAA	*/ *
*argD*-F	ATGAGCGACCTCGATTTCGT	*/ *
*argD*-R	TCATGCGTTGGAGTCGGT	*/ *
*argB*-F	GGGCACCCTAACATGACGAC	*/ *
*argB*-R	TCATTGGGCCACCTCC	*/ *
*argC*-F	ATGGCGGTCGGGACCGAG	*/ *
*argC*-R	TTAGGGTGCCCCCACGGG	*/ *
*argXW*-F	ATGACCGAATGCGTCGAGTG	*/ *
*argXW*-R	TCAGGCGGTCACCTC	*/ *
*argH*-F	ATGACCGAGGAGAGCGCTCAC	*/ *
*argH*-R	TCAGACATAGCCGTTCACCT	*/ *
*argG*-F	GATATGACCCGCGTGGCACT	*/ *
*argG*-R	TTACTGGTCGTCTCCCGTCCC	*/ *

**Table:** (a) Single growth factors used in this work and groups containing all of the growth factors belonged to the same type

Group 1: containing amino acids from 2–24	2: Asp	3: Cys	4: Ile
5: Gly	6: Glu	7: Val	8: Ser
9: Ala	10: His	11: Tyr	12: Phe
13: Met	14: Thr	15: Met	16: Trp
17: Pro	18: Leu	19: Lys	20: cystine
21: Arg	22: Asp	23: Asn	24: Gln

Group 25: containing vitamins from 26 to 35	26: vitamin H	27: vitamin C	28: vitamin B6
29: vitamin B3	30: vitamin B5	31: vitamin B4	32: vitamin B2
33: vitamin B9	34: vitamin B12	35: vitamin B1	

Group 36: containing dNTPs from 37 to 43	37: dATP	38: dGTP	39: dTTp
40: dCTP	41: dUTP	42: xanthine	43: hypoxanthine

**Table:** (b) Different combination groups containing amino acids

	Group A	Group B	Group C	Group D	Group E
Group F	9: Ala	21: Arg	23: Asn	22: Asp	3: Cys
Group G	6: Glu	24: Gln	5: Gly	10: His	4: Ile
Group H	18: Leu	19: Lys	15: Met	12: Phe	17: Pro
Group I	8: Ser	14: Thr	16: Trp	11: Tyr	7: Val

**Table:** (c) Different combination groups containing vitamins

	Group I	Group II	Group III	
Group IV	32: vitamin B2	33: vitamin B9	31: vitamin B4	35: vitamin B1
Group V	28: vitamin B6	27: vitamin C	29: vitamin B3	35: vitamin B1
Group VI	30: vitamin B5	34: vitamin B12	26: vitamin H	35: vitamin B1

**Table:** (d) Different combination groups containing purine and pyrimidine

	Group X1	Group X2	Group X3
Group X4	41: dUTP	37: dATP	39: dTTP
Group X5	42: xanthine	43: hypoxanthine	40: dCTP
	38: dGTP	38: dGTP	38: dGTP

**Table:** (e) The growth of the four auxotrophic strains on MM medium supplied with different nutrients

Supplied nutrients in MM medium	J7-2-1	J7-2-22	J7-2-26	J7-2-52
MM medium	N^a^	N	N	N
MGM medium	Y^b^	Y	Y	Y
Group ALL	Y	Y	Y	Y
Group 1	Y	Y	Y	Y
Group 25	N	N	N	N
Group 36	N	N	N	N
Group A	Y	N	N	N
Group B	N	Y	Y	Y
Group F	N	Y	N	Y
Group H	Y	N	Y	N
Group I	N	I^c^	N	N
18	Y	N	N	N
19	N	N	Y	N
21	N	Y	N	Y
Other nutrients	N	N	N	N

^a^N: the growth of the auxotrophs was not detected. ^b^Y: the growth of the auxotrophs was detected. ^c^I: an inhibition zone was detected.

**Table 4:** Molecular characterization of the auxotrophic mutants by analyzing relevant genes in the corresponding amino acid biosynthetic pathways.

Auxotrophic strains	Phenotype	Gene	Gene number	Notation	Length (bp)	Mutation status	
J7-2-1	Leu^−^	*leuA *	NJ7_G1272	2-Isopropylmalate synthase	831	None	
		*leuC *	NJ7_G1748	3-Isopropylmalate dehydratase, large subunit	1388	None	
		*leuD *	NJ7_G1749	3-Isopropylmalate dehydratase, small subunit	837	None	
		*leuB *	NJ7_G1752	3-Isopropylmalate dehydrogenase	768	12 bp fragment deletion from 690 to 701 bp	

J7-2-26	Lys^−^	*dapA *	NJ7_G4051	Dihydrodipicolinate synthase	951	None	
		*dapF *	NJ7_G4046	Diaminopimelate epimerase	1639	None	
		*lysA *	NJ7_G4047	Diaminopimelate decarboxylase	1422	None	
		*dapD *	NJ7_G4049	2,3,4,5-Tetrahydropyridine-2,6-dicarboxylate N-succinyltransferase	635	18 bp deletion from 583 to 600 bp	
		*dapB *	NJ7_G4050	Dihydrodipicolinate reductase	987	None	

Auxotrophic strains	Phenotype	Gene	Gene number	Notation	Length (bp)	Mutation Status	
						J7-2-22	J7-2-52

J7-2-22 J7-2-52	Arg^−^	*argF *	NJ7_G0258	Ornithine carbamoyltransferase	921	None	None
		*argE *	NJ7_G0259	N-Acetyl-ornithine/N-acetyl-lysine deacetylase	1110	None	None
		*argD *	NJ7_G0260	Acetylornithine aminotransferase	1155	None	None
		*argB *	NJ7_G0261	Acetylglutamate kinase	870	None	None
		*argC *	NJ7_G0262	N-Acetyl-gamma-glutamyl-phosphate reductase	1077	None	1051 bp: G-A(GGG-GAG)
		*argX *	NJ7_G0263	Ribosomal protein S6 modification protein	1043	None	None
		*argW *	NJ7_G0264	Putative biosynthetic carrier protein			
		*argH *	NJ7_G0265	Argininosuccinate lyase	1539	None	None
		*argG *	NJ7_G0266	Argininosuccinate synthase	1242	None	None

**Table 5:** Percentages of spheroplasts formation and spheroplasts regenerated to wt cells.

Percentage of Spheroplasts formation	Percentage of spheroplasts regenerated to wt cells		
	Different regeneration solutions		
About 95%	H-2 medium	18% MGM medium	Regeneration solution
	About 90%	About 80%	About 20%
