# Supplementary material for: Isolation and Molecular Identification of Auxotrophic Mutants to Develop a Genetic Manipulation System for the Haloarchaeon Natrinema sp. J7-2
Source: Archaea. 2015 May 21;2015:483194. doi: 10.1155/2015/483194 (PMC4454726; doi:10.1155/2015/483194)
Supplement: Supplementary file 1 — Supplementary Figure S1: showed the growth of Natrinema sp. J7-2 on MM plate. Supplementary Figure S2: showed that in the isolation process of mutants, four strains J7-2-1, J7-2-22, J7-2-26 and J7-2-52 which grow on MGM but failed on MM plate were obtained. Supplementary Figure S3: the auxotroph of the four auxotrophic strains were reconfirmed with the same experimental procedure that they grow on MGM but failed on MM plate. Supplementary Figure S4: amplification of important genes in the pathways of the amino acid synthesis from the four auxotrophic strains were shown. Supplementary Figure S5: showed that the molecular characterization of the gene mutation status of the auxotrophic strains. Supplementary Figure S6: showed the complementation of the mutation genes by auxotrophic strains' transformation and comparison the genes of these different transformants. Supplementary Figure S7: amplification of specific fragments in J7-2-1/ pUC19-HM1752-amyH transformants conferring amylase activity were shown. [file 483194.f1.pdf]

Isolation and molecular identification of auxotrophic mutants to develop a genetic manipulation system for the haloarchaeon *Natrinema* sp. J7-2

Jie Lv[a,b], Shuai Wang[a], Yuchen Wang[a], Yuping Huang[a], Xiangdong Chen[a,c]*

[a] State Key Laboratory of Virology, College of Life Sciences, Wuhan University, Wuhan 430072, China

[b] Henan Academy of Medical and Pharmaceutical Sciences (Zhengzhou University), Zhengzhou 450052, China

[c] Hubei Provincial Cooperative Innovation Center of Industrial Fermentation, Wuhan 430072, China

* Corresponding author.

Address: State Key Laboratory of Virology, College of Life Sciences, Wuhan University, Wuhan 430072, China

Telephone number: +86 027 68754533

Fax: +86 27 68754533.

E-mail address: xdchen@whu.edu.cn.

Supporting Information

Supporting figures

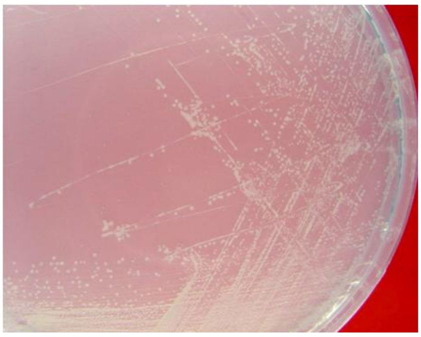

Fig. S1 Growth of *Natrinema* sp. J7-2 on MM plate
Natrinema sp. J7-2 was tested that it could grow on MM plate.

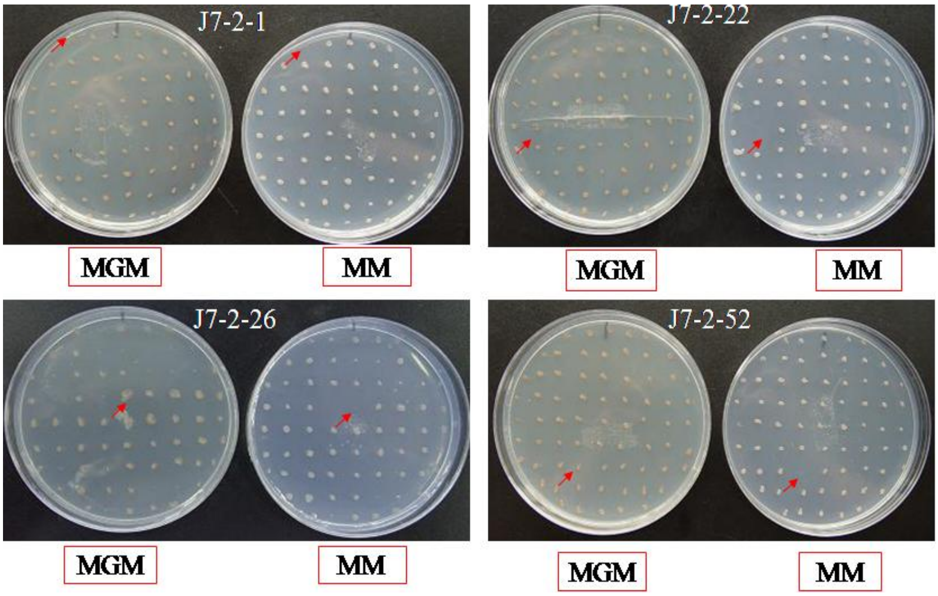

Fig. S2 Four auxotrophic strains selected on the MGM and MM plates

Four strains J7-2-1, J7-2-22, J7-2-26 and J7-2-52 which grow on MGM but failed on MM plate were obtained.

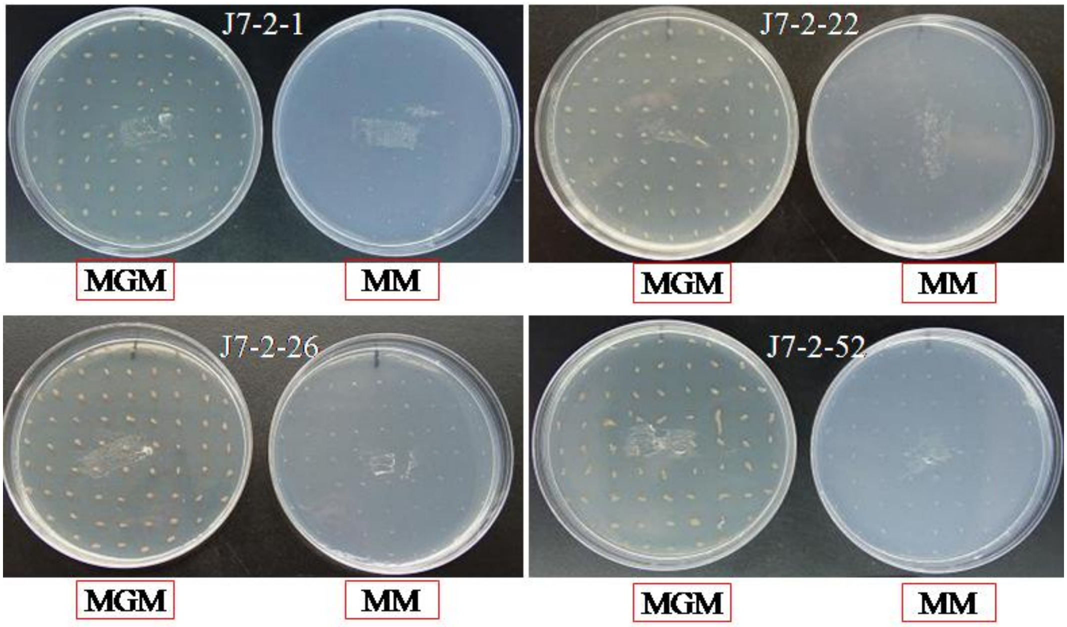

Fig. S3 Reconfirmation of four auxotrophic strains on the MGM and MM plates

The auxotroph of the four auxotrophic strains were reconfirmed with the same experimental procedure that they grow on MGM but failed on MM plate.

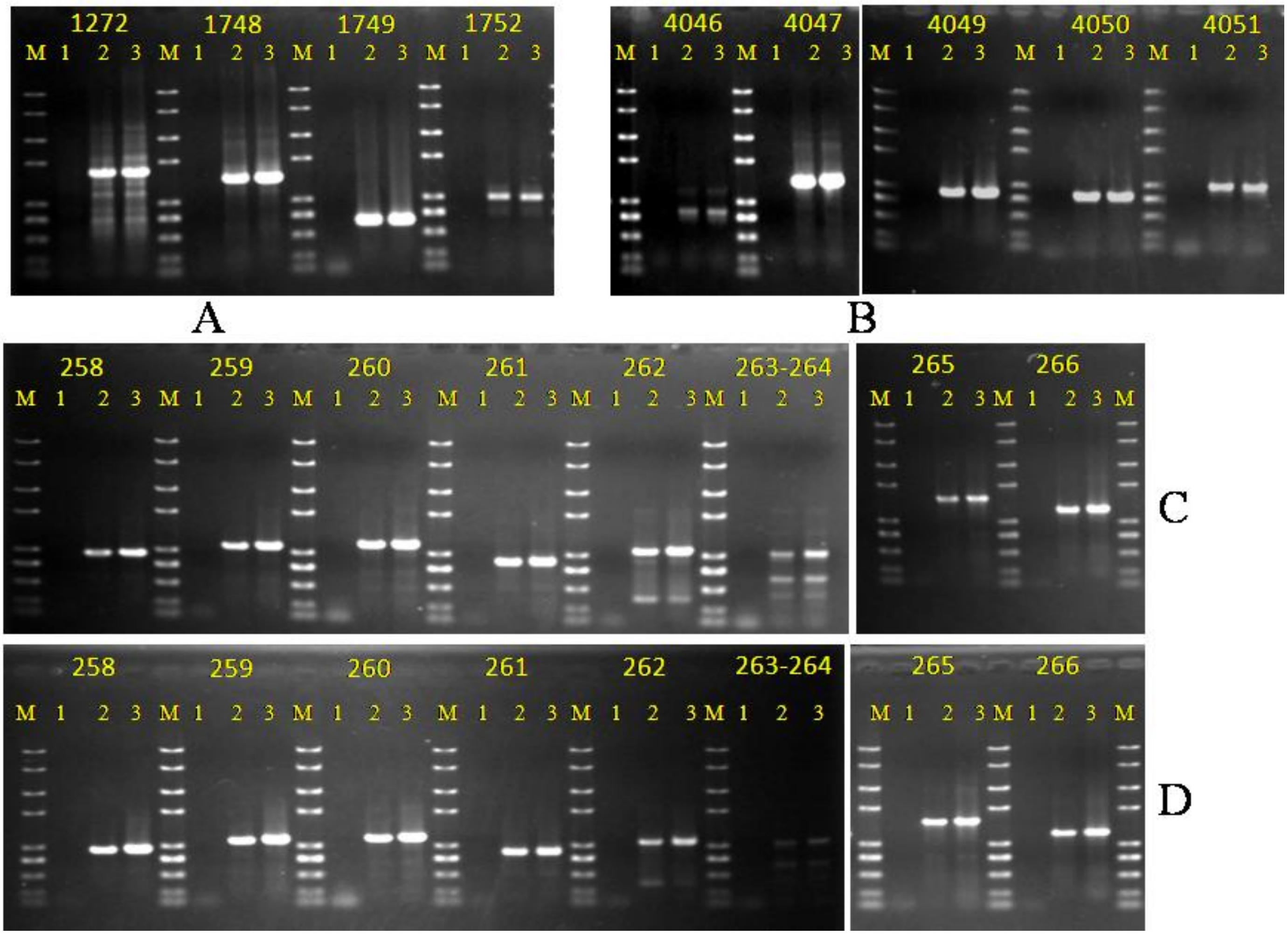

Fig. S4 Amplification of important genes in the pathways of the amino acid synthesis from the four auxotrophic strains

M: DNA Marker, 8000 bp, 5000 bp, 3000 bp, 2000 bp, 1000 bp, 750 bp, 500 bp, 250 bp, 100 bp. Lane 1, 2 and 3 between two M were fragments amplification products of corresponding genes with the same primers which were shown on the top of the lanes. DNA templates are: lane 1. H_2O as negative control, lane 2. J7-2 genome as positive control, lane 3. Genome of corresponding auxotrophs.

A. Amplification of four important genes in the pathways of leucin from J7-2-1. 1272 represent as primers of *leuA* gene, 1748 represent as primers of *leuC* gene, 1749 represent as primers of *leuD* gene, 1752 represent as primers of *leuB* gene.

B. Amplification of five important genes in the pathways of lysine from J7-2-26. 4046 represent as primers of *dapF* gene, 4047 represent as primers of *lysA* gene, 4049 represent as primers of *dapD* gene, 4050 represent as primers of *dapB* gene, 4051 represent as primers of *dapA* gene.

C and D. Amplification of nine important genes in the pathways of arginine from J7-2-22 and J7-2-52, respectively. 258 represent as primers of *argF* gene, 259 represent as primers of *argE* gene, 260 represent as primers of *argD* gene, 261 represent as primers of *argB* gene, 262 represent as primers of *argC* gene. 263-264 represent as primers of *argX* gene, 265 represent as primers of *argH* gene, 266 represent as primers of *argG* gene, 4045 represent as primers of *argE* gene.

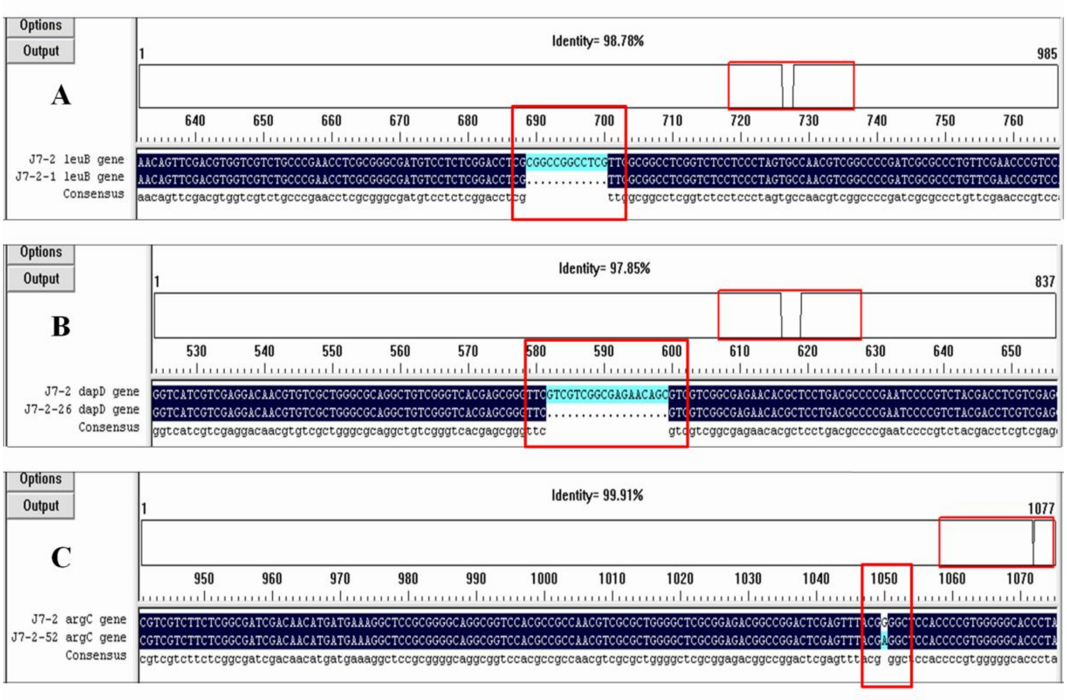

Fig. S5. Molecular characterization of the gene mutation status of the auxotrophic strains.

Some important genes in the pathways of the corresponding amino acid synthesis (Table 4) were picked out and analyzed respectively by PCR and DNA sequencing to find the gene mutations of the auxotrophic mutants. And these picked out genes of wild type strain were also amplified and sequenced as a control. Every mutant genes of the auxotrophs were determined by using DNAMAN processing software to compare of the corresponding DNA sequencing results between the wild type strain and the auxotrophs. As shown in here, *leuB* gene was the mutant gene for J7-2-1, *dapD* gene was for J7-2-26 and *argC* gene was for J7-2-52. All results in this experiment were repeated for at least three times.

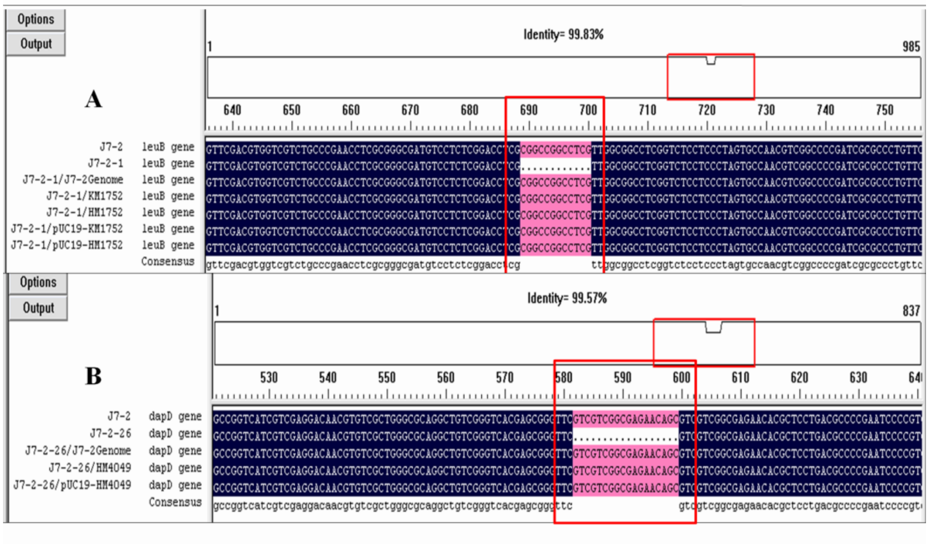

Fig. S6. Complementation of the mutation genes by auxotrophic strains' transformation and comparison the genes of these different transformants.

The mutant strain J7-2-1 was transformed with different DNA (J7-2 genome, KM1752, HM1752, pUC19-KM1752 and pUC19-HM1752). The corresponding *leuB* gene of these transformants were compared

with the wild type strain J7-2 and the mutant strain J7-2-1. And the result was shown in A.

The mutant strain J7-2-26 was transformed with different DNA (J7-2 genome, HM4049 and pUC19-HM4049). The corresponding *dapD* gene of these transformants were compared with the wild type strain J7-2 and the mutant strain J7-2-26. And the result was shown in B.

A, The mutation site (690-701, a 12bp fragment deletion) of *leuB* gene in J7-2-1 was complemented by transforming J7-2 genome, KM1752, HM1752, pUC19-KM1752 and pUC19-HM1752 into the auxotrophic strain J7-2-1. B, The mutation site (583-600, an 18bp fragment deletion) of *dapD* gene in J7-2-26 was complemented by transforming J7-2 genome, HM4049 and pUC19-HM4049 into the auxotrophic strain J7-2-26.

KM1752, HM1752, pUC19-KM1752, pUC19-HM1752, HM4049, pUC19-HM4049: please refer to Table1.

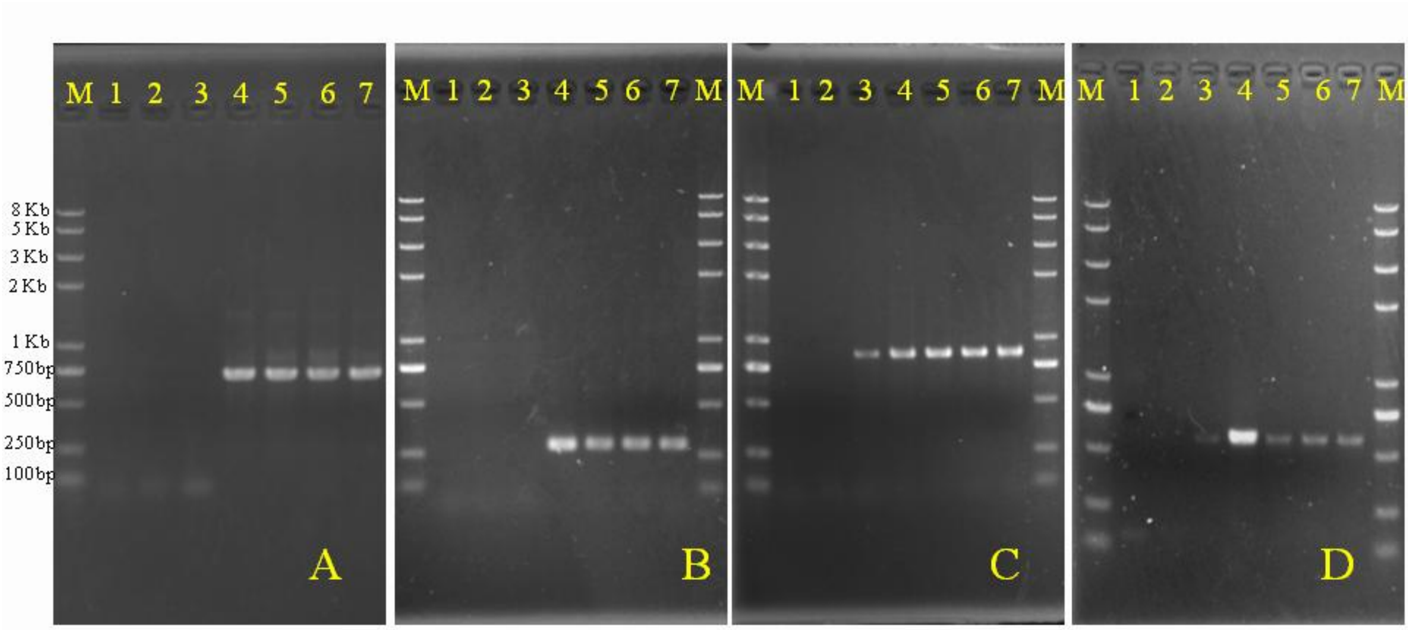

Fig. S7 Amplification of specific fragments in J7-2-1/ pUC19-HM1752-amyH transformants conferring amylase activity

J7-2-1/ pUC19-HM1752-amyH transformants conferring amylase activity were tested for other specific fragments except the *leuB* gene (1752 gene). A. Amplification of specific fragment within *amyH* gene of plasmid pUC19-HM1752-amyH. B. Amplification of specific fragment within *lacZ* gene of plasmid pUC19-HM1752-amyH. C. Amplification of specific fragment within Ampicillin resistance gene of plasmid pUC19-HM1752-amyH. D. Amplification of specific fragment within ColE1 replication region of plasmid pUC19-HM1752-amyH. In A, B, C and D. M. DNA Marker with 8 Kb, 5 Kb, 3 Kb, 2 Kb, 1 Kb, 750 bp, 500 bp, 250 bp, 100 bp, 1. Negative control with ddH2O as template. 2. Negative control with J7-2-1 as template. 3. Negative control with J7-2-1/ pUC19-HM1752-amyH transformant without amylase activity as template. 4. Positive control with plasmid pUC19-HM1752-amyH as template. 5-7. Samples with J7-2-1/ pUC19-HM1752-amyH transformant conferring amylase activity as template.
